# Editorial: Management of Patent Ductus Arteriosus in Preterm Infants

**DOI:** 10.3389/fped.2021.681393

**Published:** 2021-04-14

**Authors:** Begüm Atasay, Ömer Erdeve, Hannes Sallmon, Yogen Singh

**Affiliations:** ^1^Division of Neonatology, Department of Pediatrics, Ankara University School of Medicine, Ankara, Turkey; ^2^Department of Pediatric Cardiology, Charité - Universitätsmedizin Berlin, Berlin, Germany; ^3^Department of Congenital Heart Disease, Pediatric Cardiology, Deutsches Herzzentrum Berlin (DHZB), Berlin, Germany; ^4^Department of Neonatology and Pediatric Cardiology, Cambridge University Hospitals, Cambridge, United Kingdom; ^5^University of Cambridge School of Clinical Medicine, Cambridge, United Kingdom

**Keywords:** neonate, hemodynamics, very low birth weight, ibuprofen, indomethacin, catheter intervention, surgical ligation

Since the first known description of the human ductus arteriosus by Galen almost 2,000 years ago, basic scientists and medical professionals have extensively researched the ductus arteriosus (1, 2). In 1938, surgical patent ductus arteriosus (PDA) closure was the first corrective surgery performed for congenital heart disease by Robert E. Gross in Boston. Furthermore, interventional transcatheter PDA closure was described in 1967 by Werner Porstmann at the Charité Hospital in Berlin (1–3). In addition, the pivotal roles of prostaglandins and oxygen and their mechanisms in mediating DA closure were uncovered during the 1970's, which led to the introduction of pharmacological therapies for preterm infants with PDA (1–3). However, despite over 15,000 Medline-listed publications related to the ductus (4), the PDA of preterm infants remains an elusive condition that still challenges neonatologists and pediatric cardiologists today. Specifically, there is significant uncertainty on when and how ductus arteriosus closure in preterm infants should be attempted (5, 6). Due to a high spontaneous closure rate even in very immature preterm infants, an expectant approach may be justified in numerous cases (7). On the other hand, certain selected infants with hemodynamically significant PDA (hsPDA) may benefit from intervention, be it pharmacological, interventional, or surgical (8, 9). However, there is no consensus on the clinical and echocardiographic definition of hsPDA (10). In addition, although a hsPDA may have an impact on common neonatal morbidities such as bronchopulmonary dysplasia (BPD), pulmonary and intraventricular hemorrhage (IVH), necrotizing enterocolitis (NEC), retinopathy of prematurity, neurodevelopmental impairment, and eventually mortality, clear evidence for a causal relationship between hsPDA and these conditions is scarce (5–10). Within this Research Topic we present a collection of 24 articles, including 13 original articles, 6 systematic reviews/meta-analyses, and 6 review articles, that cover different aspects of PDA in preterm infants.

First, in a comprehensive review article, Ovali provides an overview on molecular and mechanical mechanisms that regulate ductus arteriosus closure in human preterm infants. Besides a detailed description of biochemical and molecular pathways involved in physiological ductus arteriosus closure, the author also describes signaling and cellular pathways that contribute to persistently PDA in preterm infants. In their mini-review and systematic review articles, Sallmon et al. and Gonzalález-Luis et al. specifically focus on the controversial role of platelets in human preterm ductus arteriosus closure. Their combined evidence suggests that low platelet counts are associated with PDA in preterm infants, while the clinical significance of these findings is still unclear. The meta-analysis by Liu et al. further explores risk and outcome factors that are associated with PDA in preterm infants. The authors found significant associations between PDA and immaturity, chorioamnionitis, sepsis and respiratory distress, but also between PDA and BPD, IVH and NEC, among others. However, the causality between those associations remains unclear. Canpolat et al. present additional data that suggest that surfactant application beyond the first 2 h of life is associated with a higher incidence of PDA as compared to earlier surfactant administration.

In the next article, Singh et al. provide an overview on the echocardiographic diagnosis and hemodynamic evaluation of PDA with a special focus on extremely low gestational age newborn infants. In a retrospective cohort study of preterm infants, Choi et al. further explore the relationship between echocardiographically determined ductal size, markers of shunt volume, and postnatal age. They report that ductal size within the first 3 days of life cannot adequately predict shunt magnitude, thus highlighting the need for a more complex echocardiographic evaluation of the rapidly evolving hemodynamic changes that occur during the first few days of life.

The important topic of spontaneous PDA closure is covered by a systemic review article by de Klerk et al.. In another original article, Bravo et al. describe a predictive model for spontaneous PDA closure, which is based on echocardiography and gestational age. In addition, Gonen et al. present their pilot study on a scoring system for early prediction of hsPDA development, which is entirely based on clinical criteria without echocardiographic examination. Three further original articles by Huizing et al., Coviello et al., and Bardanzellu et al. examine the associations between plasma amino acids, isoprostanes, and urinary metabolomics and PDA, respectively. Beyond, exploring the potential use of those substances as early biomarkers, these investigations may also stipulate further mechanistic research on the role of certain metabolites in neonatal PDA biology.

The following articles focus on different aspects of treatment in preterm infants with PDA. Nowadays, several clinicians prefer a conservative management approach in most infants with PDA. Sung et al. provide an overview on this option with a special focus on extremely immature infants. In their meta-analysis, Hundscheid et al. compare outcome measures between retrospective and randomized controlled trial data on conservative PDA management. The authors conclude that there still is a lack of high-quality studies on conservative PDA management. Likewise, a meta-analysis by Jansen et al. which focusses on factors associated with benefit of PDA treatment, acknowledges a low quality of available evidence. In their analysis, the only benefit of PDA treatment was a reduction in IVH rate in certain infants, while other outcome parameters were not influenced by PDA treatment. In a prospective, observational cohort study comparing conservative and medical PDA management, Okulu et al. fail to demonstrate any association between PDA treatment and outcome improvement. Of note, the authors even observed higher rates of BPD and mortality in infants treated before the first 7 days of life as compared to those treated later and those who were managed conservatively. Two further original articles focus on different approaches to identify at-risk infants, thereby limiting the potential exposure to PDA therapies. Firstly, Terrin et al. present their initial results on echocardiography-guided PDA therapy and, secondly, Ibrahim et al. report on their experience after introduction of a selective treatment approach limited to high-risk infants ≤ 800 g or < 27 weeks of gestational age.

Next, Parkerson et al. present an excellent overview on contemporary PDA-management strategies based on their pioneer role in establishing transcatheter PDA closure as an alternative therapeutic option in very immature infants. Besides interventional approaches, the authors also cover conservative, pharmacologic and surgical treatment options. Interventional PDA closure in extremely premature infants is further discussed in detail in a review article by Fraisse et al. who advocate for a validation of the initially promising results on interventional PDA closure by randomized controlled trials. While transcatheter PDA closure has been approved for infants ≥ 700 g and after 3 days of birth, surgical PDA ligation may still be required in smaller infants and in PDA which are not suitable for interventional closure. Olsson et al. present their results from a matched-cohort study on extremely preterm infants with and without PDA ligation. They show that infants in the ligated group exhibited a higher rate of severe BPD, but did not differ otherwise from the non-ligated group. Furthermore, Yang et al. report on their initial experience with PDA ligation in infants with a bidirectional shunt pattern, traditionally considered a contraindication for PDA closure. They suggest that selected infants with bidirectional shunt may benefit from PDA closure, although a higher perioperative risk needs to be weighed against the potential benefits of closure. Finally, Engeseth et al. show that infants who underwent surgical PDA ligation showed a higher incidence of exercise-induced breathing and voice problems during mid-childhood as compared to infants without PDA surgery.

In conclusion, despite an improved pathophysiologic understanding of PDA, the articles presented in this Research Topic of *Frontiers in Pediatrics* illustrate that several aspects of PDA management in preterm infants are still controversial. While a growing body of evidence suggests that a conservative approach is safe and efficient in most infants, the identification of selected at-risk patients who likely benefit from treatment is of utmost importance in order to provide tailored-care (5–9). Careful clinical and echocardiographic examinations are required to understand the current hemodynamic situation of each patient at each given time point. However, standardized validated protocols for hsPDA assessment are still lacking (5, 10). Furthermore, the optimal timing of treatment and the choice of the therapeutic modality (conservative, pharmacologic, interventional, surgical) are still subject to debate (5–8). Thus, there is a need for additional randomized-controlled trials that specifically address these issues in the high-risk population of extremely immature infants with PDA (9). In the meantime, adopting an individualized approach based upon clinical and echocardiography parameters seems sensible and appropriate. We hope that the collection of articles presented herein will further stipulate research efforts in order to optimize the care of preterm infants with PDA.

## Author Contributions

All authors listed have made a substantial, direct and intellectual contribution to the work, and approved it for publication.

## Conflict of Interest

The authors declare that the research was conducted in the absence of any commercial or financial relationships that could be construed as a potential conflict of interest.
